# Hypothalamic overexpression of mutant huntingtin causes dysregulation of brown adipose tissue

**DOI:** 10.1038/srep14598

**Published:** 2015-09-30

**Authors:** Rana Soylu-Kucharz, Natalie Adlesic, Barbara Baldo, Deniz Kirik, Åsa Petersén

**Affiliations:** 1 Translational Neuroendocrine Research Unit, Department of Experimental Medical Sciences, Lund University, Sweden; 2 Brain Repair and Imaging in Neural Systems (B.R.A.I.N.S.) Unit, Department of Experimental Medical Sciences Lund University, Sweden

## Abstract

Expression of mutant huntingtin (htt) protein has been shown to cause metabolic imbalance in animal models of Huntington disease (HD). The pathways involved are not fully understood but dysfunction of both the hypothalamus and brown adipose tissue (BAT) has been implicated. Here we show that targeted expression of mutant HTT in the hypothalamus leads to loss of the A13 dopaminergic cell group located in the zona incerta and reduced mRNA expression of neuropeptide Y1 receptor in the hypothalamus. Furthermore, this is accompanied by downregulation of uncoupling protein 1 expression and PPARγ coactivator-1 alpha in BAT and a rapid body weight gain. Taken together, our data might provide a mechanistic link between expression of mutant HTT, reduced activity of a hypothalamic dopaminergic pathway and dysfunction of BAT and in part explain the development of an obese phenotype in HD mouse models.

The hypothalamus is the master regulator of homeostatic energy metabolism. Previous studies have dissected out pathways within the hypothalamus that may have an important role in the control of body weight^1^,^2^,^3^. While midbrain dopaminergic neurons are known to regulate reward and motivational aspects of feeding^4^,^5^, reduced dopaminergic tone in their hypothalamic counterparts referred to as the A12 group in the arcuate area, the A13 group in the zona incerta and the A14 group in the periventricular area in mice has been implicated in the development of obesity^6^,^7^,^8^. The underlying mechanisms for the dopaminergic control of body weight are not known^9^,^10^.

The disease-causing form of huntingtin (HTT) is the culprit of the neurodegenerative Huntington disease (HD)^11^. Importantly, metabolic dysfunction and hypothalamic changes have emerged as important aspects of non-motor symptoms in HD^12^,^13^. Both the mutant and normal forms of HTT have been suggested to exert effects on metabolic regulation *in vitro* and *in vivo*^14^. Genetic mouse models of HD with ubiquitous expression of mutant HTT display metabolic dysfunction but vary in the phenotype with either increased or decreased body weight compared to their wild-type littermates^15^,^16^,^17^,^18^,^19^,^20^,^21^. It is not known why there are differences in the metabolic phenotype between the models. We have recently linked hypothalamic overexpression of the mutant HTT protein to dysregulation of body weight. Selective expression of the mutant form of HTT in the hypothalamus by using recombinant adeno-associated viral (rAAV) vectors led to the development of a severe metabolic phenotype with obesity accompanied by leptin and insulin resistance in mice. Moreover, inactivation of mutant HTT in the hypothalamus of a transgenic HD mouse prevented the development of the obese metabolic phenotype^16^. Interestingly, hypoactivity of brown adipose tissue (BAT) with down-regulation of peroxisome proliferator-activated receptor gamma coactivator 1-alpha (PGC1-α) as well as uncoupling protein 1 (UCP1), have been found in mouse models of HD^22^,^23^,^24^,^25^,^26^,^27^,^28^. These alterations of key regulators of adaptive thermogenesis were found in mouse models with ubiquitous expression of mutant HTT, and were linked to metabolic defects^21^. Yet, the key pathways that mediate the metabolic effects of HTT have not been identified.

Recently, a novel signaling pathway regulated by neuropeptide Y (NPY), a well-known inducer of feeding was proposed. It was demonstrated that NPY from the arcuate nucleus negatively acts on the tyrosine hydroxylase (TH)-expressing population in the A13 area, which in turn regulates BAT^23^. In the study by Shi *et al.* the upregulation of NPY led to reduced energy metabolism and obesity via reduced TH expression in the hypothalamus and decreased levels of UCP1^23^. Based on the neurotoxicity of mutant HTT, we hypothesized that the protein would directly act on the hypothalamic dopaminergic population and thereby lead to obesity via hypofunction of BAT. In this study, we therefore investigated how this metabolic circuitry was affected by targeted expression of HTT in the hypothalamus.

## Results and Discussion

### Expression of mutant HTT reduces the number of TH-expressing neurons in the A13 zona incerta area of the hypothalamus

**Figure 1:**
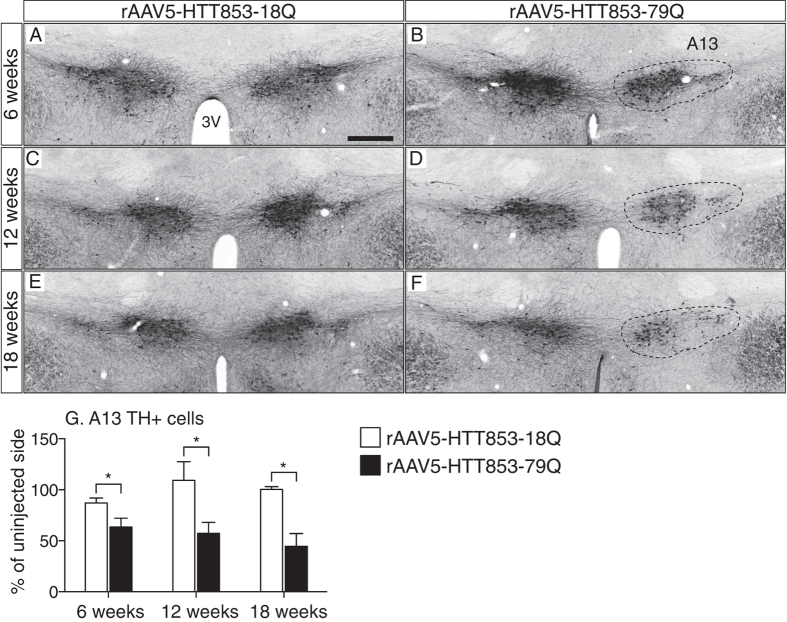
Early loss of A13 TH-immunopositive cells in the mutant HTT-expressing hypothalamus. (**A–F**) Representative images of TH immunohistochemistry showing the population of A13 TH+ cells in the hypothalamus after unilateral injections of either rAAV5-HTT853-18Q or rAAV5-HTT853-79Q at 6 weeks (**A,B**); 12 weeks (**C,D)** and 18 weeks post-injection (**E,F**). **(G**) Stereological estimation of the number of analysis of TH+ cells in the A13 area at 6; 12; and 18 weeks post-injection. Data is presented as a percentage of A13 TH+ neurons in relation to the uninjected side (n = 4–6 animals/group, *p < 0.05, unpaired t-test). 3V = 3^rd^ ventricle. Scale bar in all panels = 200 μm.

First, we performed immunohistochemistry for TH to investigate whether selective expression of mutant HTT in the hypothalamus has an effect on the A13 group in the zona incerta (Fig. 1A–F). For this purpose, we used brain tissue from wild-type mice that were stereotactically injected with rAAV serotype 5 (rAAV5) vectors expressing the first 853 amino acids of HTT with 79Q (rAAV5-HTT853-79Q; disease causing mutant HTT) or 18Q (rAAV5-HTT853-18Q; wild-type variant) into the hypothalamus. We have previously shown that expression of 79Q in the hypothalamus led to rapid development of a severe metabolic phenotype^16^. Stereological estimates of the total number of A13 dopaminergic neurons present on the viral vector injected side revealed a significant loss in the 79Q group already 6 weeks post-injection compared to the uninjected side. Moreover, this loss in 79Q group coincided with weight gain onset, as compared to the 18Q animals^16^. Loss of TH positive (TH+) neurons in the A13 area of 79Q animals was estimated to be 37 ± 9%, 43 ± 11% and 55 ± 13% (at 6, 12 and 18 weeks post-injection, respectively) as compared to the uninjected side. Notably, this effect appeared to be specific to the mutant protein as no such reduction in TH+ numbers was present in the 18Q group (Fig. 1G). Hence, the TH+ population in the A13 area of the hypothalamus was severely affected by expression of mutant HTT.

### Targeting of the A12, A13 and A14 groups and effects of long term transgene expression after injections of rAAV5 vectors into the hypothalamus

**Figure 2:**
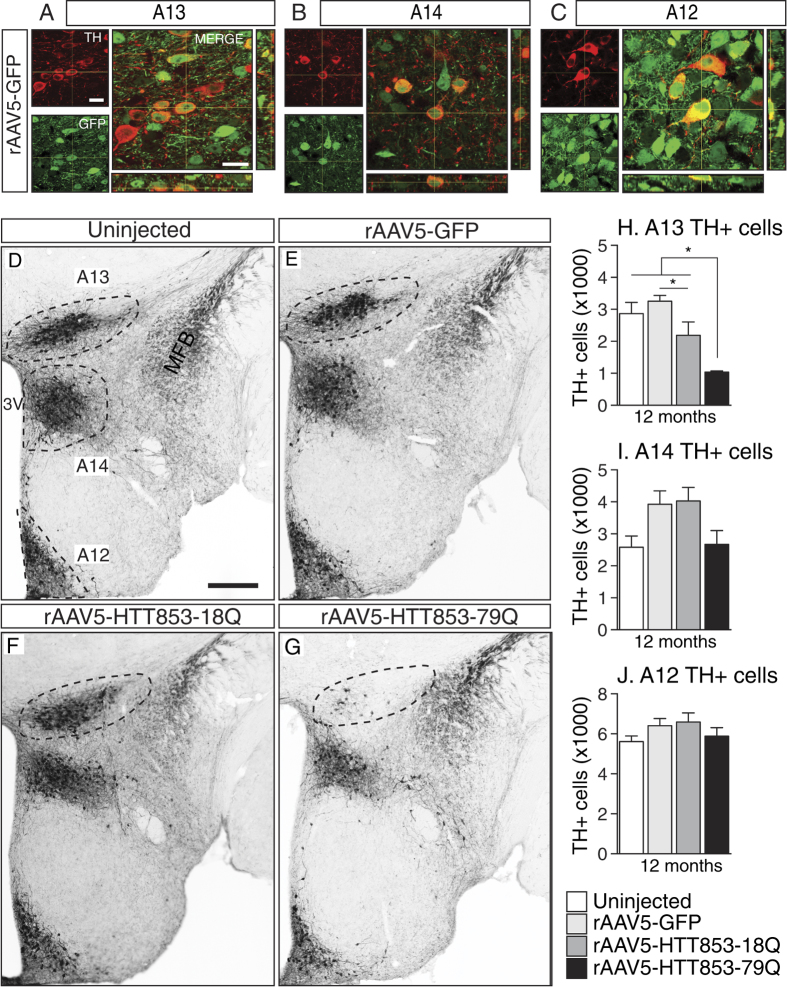
Targeting of different TH cell populations and stereological assessment of dopaminergic cells in the hypothalamus at 12 months post-injection. (**A–C**) The orthogonal projections of Z-stack image series show the co-localization of TH+ cells (red) with GFP transgene (green) in the A13, A14 and A12 hypothalamic regions of animals injected with rAAV5-GFP vector. (**D–G**) Photomicrographs representing the A13, A12, and A14 TH+ cell populations in the hypothalamus at the 12 month time-point. (**H–J**) Stereological estimation of the number of A13, A14 and A12 TH+ cells in the hypothalamus (n = 7–11/group, *p < 0.05, one-way ANOVA followed by Tukey’s posthoc test). Data is presented as mean ± SEM. 3V = 3^rd^ ventricle; MFB = medial forebrain bundle. Scale bar in A–C = 20 μm; D–G = 200 μm.

Next, we wanted to confirm that the A12, A13 and A14 cell groups were all transfected by the rAAV5 vectors and investigate whether there was a selective sensitivity of the A13 group to expression of mutant HTT. As wild-type HTT has been shown to have metabolic effects^20^,^29^, we were also interested in studying the long-term consequences of wild-type HTT expression in the hypothalamus. To control for aberrant protein overexpression, we included a group injected with a vector encoding the green fluorescent protein (GFP) that also served as an extra control group. The animals were kept up to 12 months post-injection, and the pattern of expression was analyzed using confocal microscopy. The fluorescence imaging of the GFP expressing brain sections showed that all three dopaminergic cell populations in the hypothalamus were transfected (Fig. 2A–C).

**Figure 3:**
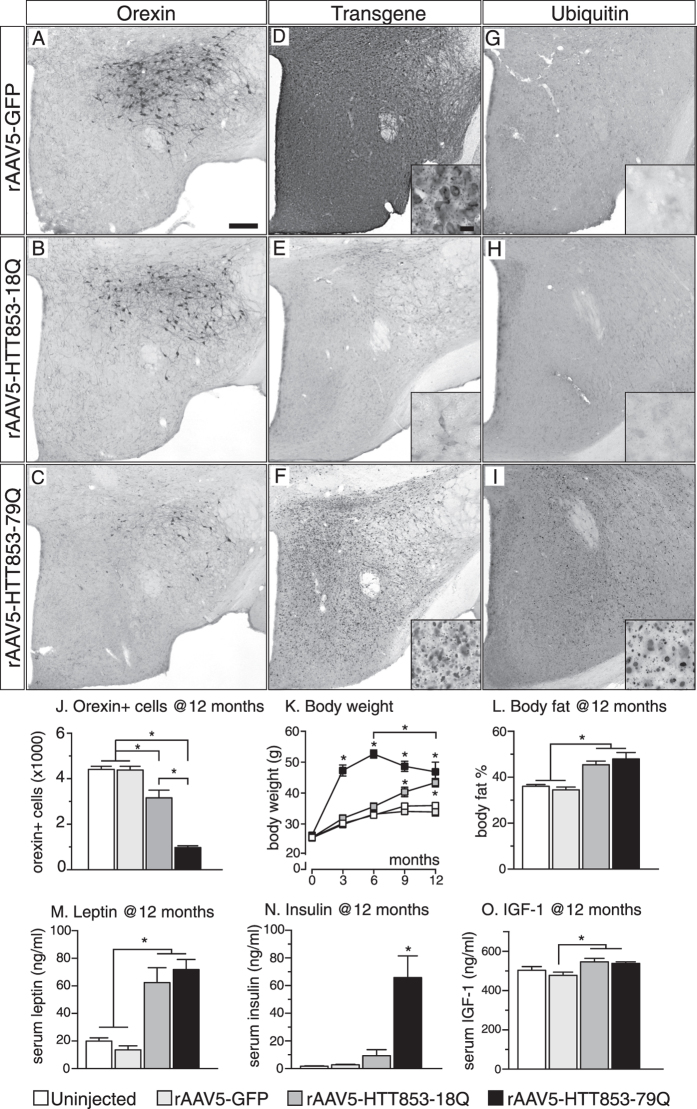
The hallmarks of brain and systemic metabolic changes in mice selectively expressing the 79Q and the 18Q HTT variants in the hypothalamus. (**A–C**) Representative photomicrographs of hypothalami sections processed for orexin immunohistochemistry from mice 12 months post-injection. (**D**) Representative images from GFP immunohistochemistry showing the coverage of transgene expression in the hypothalamus. (**E,F**) Anti-huntingtin immunohistochemistry (sc87-67) demonstrating diffuse cytoplasmic staining in the 18Q group in contrast to the HTT inclusion formation in the 79Q group. (**G,H**) Anti-ubiquitin immunohistochemistry demonstrating absence of inclusion formation in GFP and 18Q group; and widespread ubiquitin positive inclusion formation in the 79Q group at 12 months (**I**). (**J**) Stereological assessment of the number of orexin+ cells at 12 months post-injection in the hypothalamus (n = 7–11/group). (**K**) Distinct changes in body weight over time monitored up to 12 months post-injection in animals expressing different HTT variants compared to controls (n = 20/group until 6 months, then n = 13–20/group). (**L**) Assessment with DEXA scan to show differences in percentage total body fat content between groups at 12 months (n = 10–19/group). (**M–O**) Evaluation of leptin, insulin and IGF-1 blood serum levels (n = 9–10/group). *p < 0.05 one- or two-way ANOVA followed by Tukey’s posthoc test. Data represented as mean ± SEM. Scale bar = 200 μm in (**A–I**) and 20 μm in high magnification images.

Stereological analyses of the numbers of TH+ neurons in the A12-A14 groups confirmed that the A13 group was selectively affected by the expression of mutant HTT (Fig. 2D–J). The A12 and A14 groups remained unaffected despite the long-term follow-up and persistence of transgene expression (Fig. 2I,J). This data shows that the A13 TH population is highly vulnerable to overexpression of mutant HTT, a property not shared by the two other neighboring dopaminergic cell populations. Interestingly, at 12 months post-injection there was a measureable effect of wild-type HTT overexpression on the A13 cell group (Fig. 2H). The vulnerability of the A13 group to HTT overexpression mirrored the sensitivity of the orexin population, known to be affected in the HD hypothalamus^12^,^16^,^30^ (Fig. 3A–C,J).

The underlying mechanism of selective vulnerability of specific neuronal populations in HD, as well as in other neurodegenerative disorders, is not fully understood. The concept of this limited vulnerability is changing however, as more studies demonstrate pathology in other brain regions than those initially thought to be solely affected. Hypothalamic dopaminergic neurons are resistant to exposure of the neurotoxin 6-hydroxydopamine that causes massive loss of dopaminergic neurons in the substantia nigra in rats^31^. Another study demonstrated that systemic exposure to the neurotoxin MPTP causes dose-dependent loss of TH-immunoreactivity in the A13 area, similar to that in the substantia nigra and ventral tegmental area in mice^32^. Effects on A12 and A14 were not investigated in that study. In Parkinson disease, biochemical and neuroimaging studies have indicated dopaminergic dysfunction in the hypothalamic region, but these experimental approaches do not distinguish between different dopaminergic populations in the hypothalamus^33^,^34^,^35^. In the present study, we show that A13 dopaminergic neurons are selectively sensitive to the expression of mutant HTT compared to A12 and A14 neurons. These data underlie the necessity to examine postmortem material in HD patients.

### Long-term metabolic consequences of hypothalamic overexpression of wild-type HTT

In our previous study we did not see any effect of hypothalamic overexpression of a wild-type HTT fragment on metabolic parameters in mice followed up to 18 weeks post-injection^16^. In the present study, however there was a delayed but persistent body weight increase in the 18Q group that resulted in a similar body weight gain as in 79Q with increased body fat by 12 months of age (Fig. 3K,L). As mice from both groups display orexin and TH A13 loss, although to a different degree, it is possible that dysfunction of these two neuronal populations is involved in the long-term effects exerted by the HTT protein on metabolic control. Importantly, mice with 79Q displayed a significantly different trajectory of the body weight gain than mice with 18Q (Fig. 3K). There are several potential factors that could explain this difference.

First, the metabolic phenotypes of the two groups were not the same. The insulin levels were significantly increased in the 79Q group compared to the 18Q group at 12 months despite that levels of leptin and insulin growth factor 1 (IGF-1) were comparable (Fig. 3M–O). The faster body weight gain in the 79Q group could contribute to this phenomenon. It is also possible that the presence of HTT inclusions and/or the mutant HTT protein negatively interacts with downstream factors in the insulin signaling pathway in the hypothalamus, contributing to the increased levels of insulin. Both the Akt signaling and NF-kappaB signaling pathways, important mediators of insulin action in the CNS^36^, are downregulated in experimental models of HD and tissue from HD patients^37^,^38^,^39^. However, whether these pathways are affected in the HD hypothalamus, is as yet unknown.

Second, another important difference between the two groups is that overexpression of mutant HTT in the hypothalamus induced formation of a large number of intracellular inclusions immunopositive not only for HTT but also for ubiquitin (Fig. 3D–I), a hallmark of HD pathology^40^. No inclusions immunopositive for HTT or ubiquitin were found in the 18Q group. Notably, as intracellular inclusions in this model are present already at 6 weeks^16^ and remain in the hypothalamus at 12 months post-injection when the neurodegenerative process has taken place, it is plausible that dysfunction of inclusion-containing neurons also contribute to the rate and extent of the hypothalamic dysfunction-induced body weight gain. This is supported by the fact that formation of ubiquitin-positive aggregates in the hypothalamus has been associated with the development of an obese phenotype in mice transgenic for E4B, an ubiquitin chain elongation factor^41^.

Another interesting point is that the body weight in the 79Q group was significantly lower 12 months post-injection compared to the weight 6 months post-injection (Fig. 3K). Although these mice were not followed longer than 12 months, one may speculate that the weight loss would continue, eventually mirroring the clinical situation at end-stage^42^,^43^.

### Targeted overexpression of mutant HTT in the hypothalamus leads to dysregulation of the NPY-responsive circuitry to brown adipose tissue

**Figure 4:**
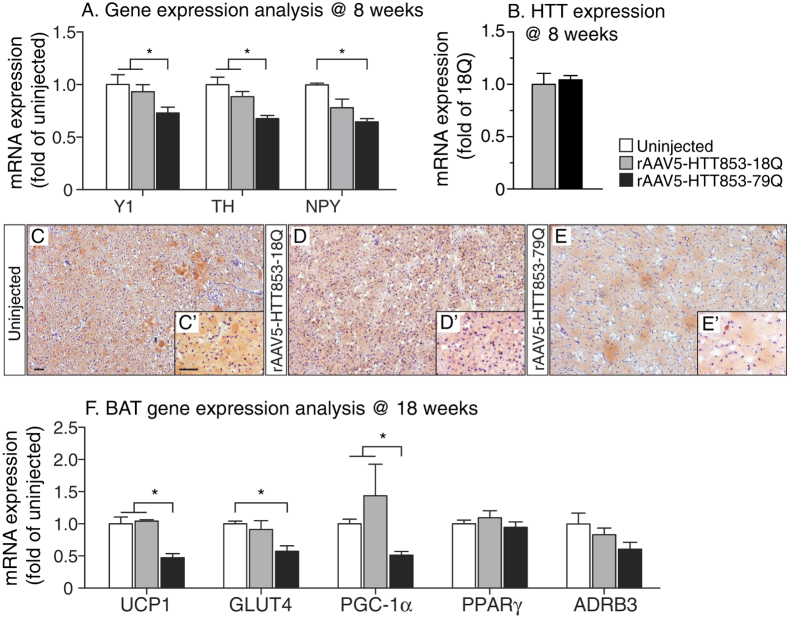
Gene expression changes in the metabolism-regulating dopaminergic circuitry to brown adipose tissue induced by mutant HTT expression in the hypothalamus. (**A**) mRNA levels of Y1, TH and NPY in the hypothalamus at 8 weeks post-injection (n = 7/group, *p < 0.05, one-way ANOVA followed by Tukey’s post-hoc test). Data was normalized to mRNA of *actb* and *gapdh* house keeping genes and presented as a fold change in relation to uninjected animals. (**B**) Similar mRNA levels of HTT853-18Q and HTT853-79Q after the injections of the viral vectors in the hypothalamus. The data is presented as fold change of HTT853-18Q (n = 5–7/group, ns, unpaired t-test). (**C–E**) Qualitative analysis of BAT at 18 weeks post-injection. The Oil Red O staining shows the increased prevalence of intracellular lipid droplets (red) and reduced number of nuclei (blue) in rAAV5-HTT853-79Q group compared to uninjected and rAAV5-HTT853-18Q animals. (**F**) Assessment of mRNA level changes in BAT in animals injected in the hypothalamus with either rAAV5-HTT853-18Q or rAAV5-HTT853-79Q. Data is presented as mean ± SEM of fold change in relation to uninjected animals after normalization to mRNA levels of *actb* and *rlp13a* housekeeping genes (n = 6/group, *p < 0.05, Kruskal-Wallis test followed by Dunn’s multiple comparison test). 3V = 3^rd^ ventricle. Scale bar = 50 μm in all panels.

Given that NPY from the arcuate nucleus negatively regulates TH expression via, as proposed previously, Y1 receptors expressed on the TH+ neurons in the A13 cell population^23^,^44^ we investigated if the effects mediated by mutant HTT included alterations in Y1 receptor mRNA levels. We found that in 79Q group the mRNA levels of both Y1 receptor and TH were significantly downregulated compared to the 18Q group (Y1: ~22% loss; TH: ~24% loss) and an uninjected control group (Y1: ~27% loss; TH: ~32% loss) (Fig. 4A). qRT-PCR analysis confirmed similar mRNA levels of HTT expression in the 79Q and 18Q groups (Fig. 4B).

Reduction of TH in the A13 hypothalamic population has been proposed to lead to reduced activity of BAT with down-regulation of UCP1, the key regulator of adaptive thermogenesis present in BAT mitochondria^23^,^45^. In addition to that, orexin has recently been found to regulate metabolism via effects on BAT differentiation and thermogenesis, and mice with orexin loss develop obesity by BAT hypoactivity^46^,^47^,^48^. Here, we show on Oil Red O staining that the BAT in the 79Q group displayed marked accumulation of lipid droplets and less cellular nuclei, both indicative of BAT hypofunction (Fig. 4C–E). qRT-PCR analysis of BAT confirmed a significant reduction in UCP1 levels by ~50% in the 79Q group compared to both the 18Q group and uninjected mice (Fig. 4F). Furthermore, the expression levels of PGC1-α, a transcriptional co-activator important for BAT thermogenesis^49^, were significantly reduced in the 79Q group compared to both the 18Q and the uninjected control group (~63%; ~47%, respectively; Fig. 4F). Interestingly, downregulation of PGC1-α in BAT has been found also in other mouse models of HD with ubiquitous expression of mutant HTT, where it has been linked to metabolic defects^21^. The level of insulin-sensitive glucose transporter GLUT4^50^ gene expression was also reduced in the 79Q group compared to the other groups. However, mRNA levels for PPARγ, that is required for adipogenesis, and ADRB3, known to regulate lipolysis and thermogenesis, were not affected^51^,^52^,^53^. Taken together, the data from the BAT analyses confirm that the activity of this tissue is affected in the predicted fashion based on the disruption of the TH-signaling pathway in the hypothalamus, here induced by expression of mutant HTT.

## Conclusions

**Figure 5:**
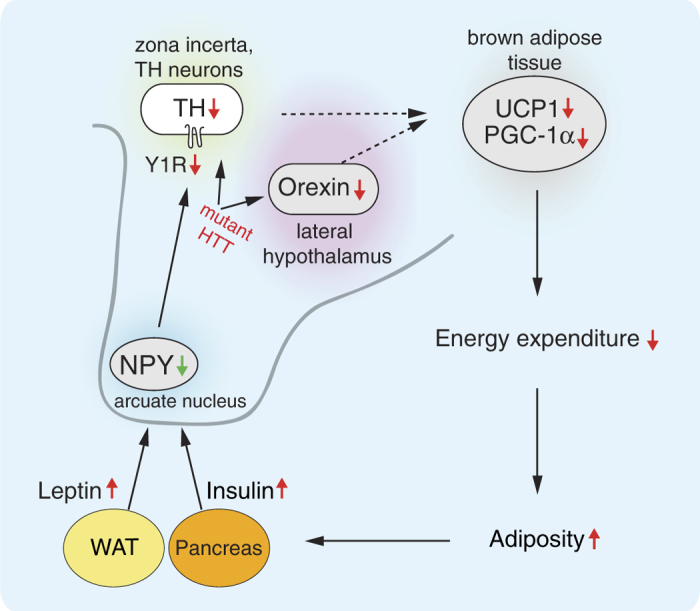
A proposed model of the effect of mutant HTT expression in the hypothalamus on the metabolic circuitry to BAT. BAT: brown adipose tissue; HTT: huntingtin; NPY: neuropeptide Y; PGC1-α: peroxisome proliferator-activated receptor gamma coactivator 1-alpha; TH: tyrosine hydroxylase; UCP1: uncoupling protein 1; WAT: white adipose tissue, Y1R: neuropeptide Y receptor Y1.

We have previously shown that overexpression of the disease causing protein HTT selectively in the hypothalamus is sufficient to induce a rapid development of a severe metabolic phenotype with leptin and insulin resistance^16^. Here we provide further insight, showing that selective hypothalamic overexpression of mutant HTT directly leads to a reduction of the TH A13 group in the zona incerta with downstream negative effects on BAT (Fig. 5). Hence, targeted expression of mutant HTT in the hypothalamus is sufficient to cause dysregulation of BAT function, previously shown to be present in mouse models with ubiquitous expression of the mutant protein^21^.

Moreover, we found that the metabolic changes are associated with increased leptin and insulin levels, which then negatively regulate NPY in the hypothalamus (Fig. 4A). Hence, although the circuitry from the periphery to the hypothalamus appears to be intact, it fails to modulate the body weight, presumably due to the effects of expression of mutant HTT directly in the A13 TH neurons. Furthermore, as this occurs in the absence of an upregulation of NPY, previously suggested to initiate this pathway in the development of obesity^23^, our results indicate that direct effects on the TH A13 population are sufficient to derail this metabolic circuitry.

The metabolic dysregulation in our model is also associated with loss of orexin, important for BAT function. Interestingly, although mutant HTT exerts rapid and strong effects on the metabolic system, the wild-type form appears to mimic the same function over time. Taken together, our data indicate that HTT regulates critical metabolic pathways from the TH A13 area and the orexin system in the hypothalamus to BAT in the periphery (Fig. 5).

## Material and Methods

### Animals

The experimental procedures performed on mice were carried out in accordance with the approved guidelines in the ethical permit approved by the Lund University Animal Welfare and Ethics committee in the Lund-Malmö region (ethical permit numbers M20-11 and M65-13). The experiments were carried out on female mice of the FVB/N strain (The Jackson Laboratories). The animals were kept at 12 hours night and day cycle with free access to water and normal chow diet.

### Adeno-associated viral vectors

In order to investigate the effect of huntingtin (HTT) expression on the hypothalamic dopaminergic circuitry we performed stereotactic injections of recombinant adeno-associated viral (rAAV) vectors expressing either a mutant or a wild-type HTT variant into the hypothalamus of mice. The viral vectors were pseudotyped rAAV2/5 vectors, where the transgene of interest was flanked by inverted terminal repeats of the AAV2 packaged in an AAV5 capsid (referred to as rAAV5 elsewhere). Each HTT insert contained the N-terminal fragment of the human HTT gene of 853 amino acids length (obtained from Dr Nicole Deglon^54^), expressed under the human Synapsin-1 (Syn-1) promoter. The wild-type variant of HTT contained 18 CAG repeats (HTT853-18Q), whereas the mutant form contained 79 CAG repeats (HTT853-79Q). Both HTT inserts and the GFP cDNA were used to generate viral vector constructs with a woodchuck hepatitis virus post-transcriptional regulatory element (WPRE) attached downstream to the respective transgene. This was followed by an early SV40 poly-A and flanked by two AAV2 ITRs sequences. The whole construct was packaged into a rAAV5 capsid. The rAAV vectors were obtained with a double-transfection method utilizing the helper plasmid encoding essential adenoviral packaging genes, as described before^55^. rAAV5 vectors expressing green fluorescent protein (GFP) under the Syn-1 promoter were used to illustrate transduction efficiencies.

### Viral vector injections

Mice of 2 months of age and weighing 20–26 g were injected in the hypothalamus either unilaterally or bilaterally with 0.5 μl/side of rAAV5-HTT853-79Q, rAAV5-HTT853-18Q or rAAV5-GFP. First mice under 2% isoflurane in oxygen/nitrous oxide (3:7) anesthesia were transferred onto a stereotactic instrument. The stereotaxic coordinates for the hypothalamic region were determined according to the 3rd edition Franklin and Paxinos brain atlas, and were 0.6 mm posterior and 0.6 mm lateral to the bregma^56^. Then the skull of the animal was thinned with dental drill at the chosen location and the thin bone flap was carefully removed leaving the dura intact. A pulled glass capillary (outer tip diameter ≈80 μm) attached to a 5 μl Hamilton syringe (Nevada, USA) was used to inject the total volume of 0.5 μl of viral vectors at the depth of 5.2 mm ventral to the dura mater. The viral vectors were delivered with 0.05 μl injections in 15 s intervals, subsequently to an initial injection of 0.1 μl of viral vector solution. Following the injection, the glass capillary was left in the target for additional 5 minutes to allow absorbance of the virus by the tissue. The vector concentrations were: rAAV5-HTT853-79Q; 1.6-2.14E × 14 genome copies (GC)/ml; rAAV5-HTT853-18Q; 1.4-2.1E × 14 GC/ml and rAAV5-GFP; 5.7Ex13 GC/ml.

### The experimental timeline

The long-term study of HTT-induced brain changes was performed on mice injected either with rAAV5-853HTT-18Q or rAAV5-853HTT-79Q variants at 2 months of age. The body weight of all animals was measured every two weeks until 12-months post-injection, when the mice were sacrificed for analysis of insulin, leptin, IGF-1 levels, and stereological assessment of TH and orexin positive cells. The control groups constituted of non-injected and rAAV5-GFP-injected animals (4 groups in total, n = 20 per group). The gene expression analysis of hypothalami from mice 8 weeks post-injection was performed on tissue isolated previously^57^. The analysis of changes in brown adipose tissue (BAT) was performed at 18 weeks post-injection and the assessment of TH+ cells at 6, 12 and 18 weeks post-injection in tissue isolated previously^16^.

### Immunohistochemistry

For the immunohistochemical analysis of brain tissue, the sodium pentobarbital-anesthetized mice were first perfused transcardially with saline and subsequently with pre-cooled 4% paraformaldehyde (PFA) at 10 ml/min rate for 10 minutes. The dissected brains were placed in 4% PFA solution at 4 °C for 24 hours, then transferred to 25% sucrose solution 4 °C for ~24 hours. From each collected brain, a series of 30 μm thick coronal sections were cut using a Microm HM450 microtome (Thermo Scientific). The sections were stored at −20 °C in an antifreeze solution (30% glycerol, 30% ethylene glycol solution in PBS) until further processing.

For the immunohistochemistry, free-floating brain sections were rinsed 3 times for 10 minutes with 0.05 M Tris-buffered saline (TBS) to remove the antifreeze solution. Following that, a quenching reaction was performed in TBS containing 3% H2O2 and 10% Methanol. The sections were pre-incubated for 1 hour at the room temperature (RT) with blocking solutions containing 5% normal goat serum and 0.25% triton-X in TBS (TBS-T). Next, the sections were left overnight at RT in primary antibody solution in TBS-T containing 5% normal goat serum. The primary antibodies dilutions were: anti-tyrosine hydroxylase (1:2000; rabbit; Pel-Freez), anti-huntingtin (sc-8767; 1:500; goat; Santa Cruz), anti-ubiquitin (1:2000; rabbit; Dako), anti-GFP (ab290; 1:30000; rabbit; Abcam), anti-orexin (1:4000; rabbit; Phoenix Pharmaceuticals). Following the primary antibody incubation, the brain sections were rinsed 3 times for 10 minutes in TBS-T and left for 1 hour in 1% bovine serum albumin (BSA) TBS-T containing secondary biotinylated antibodies (1: 200; Vector Laboratories Inc.). Next, the sections were washed with TBS-T and incubated with an avidin-biotin-peroxidase complex solution for 1 h, then rinsed with TBS-T and the staining was visualized using 3,3′-diaminobenzidine (DAB) and 0.01% H2O2 according to manufacturers instructions. Lastly, sections were mounted on chromatin-gelatin coated glass slides, dried overnight at RT at room temperature, dehydrated in increasing alcohol solutions (70%, 95%, 100%), cleared in xylene and secured for imaging with glass coverslips using Depex mounting medium (Sigma-Aldrich).

### Stereological analysis

Unbiased stereological quantification principles were implemented to estimate the numbers of TH+ cells in the A12, A13 and A14 areas as well as the numbers of orexin positive cells in the hypothalamus by using the optical dissector method^58^. The region of interest was first delineated under the 4X objective and the counting was performed using a 60X Plan-Apochromat 1.4 N.A. oil immersion objective using Nikon Eclipse 80i upright microscope equipped with an X–Y motorized stage (Märzhauser, Wetzlar, Germany); a Z-axis motor with a high precision linear encoder (Heidenhain, Traunreut, Germany). The procedure was carried out with a random start systematic sampling routine (NewCast Module; VIS software; Visiopharm A/S, Horsholm, Denmark). A PC computer controlled all three axes and the input from the digital camera. In order to minimize the coefficient of error, the sampling interval was adjusted to count at least 100 cells for each analyzed hypothalamus.

### Confocal imaging

For the confocal imaging, free-floating brain sections were incubated for 10 min in TBS with 0.05% Triton-X-100 containing 5% normal serum matched to corresponding secondary antibody. Following that, the sections were incubated overnight at RT in 1% BSA in TBS with 0.05% Triton-X-100 solution containing primary antibodies. The primary antibody dilutions were: anti-tyrosine hydroxylase (ab1542, 1:500; sheep; Chemicon) and anti-GFP (ab13970; 1:10000; chicken; Abcam). Following the rinsing in TBS-T, the sections were incubated with DyLight 488 (1:200; donkey anti-chicken, Jackson ImmunoResearch) and Cy5 (1:200; donkey anti-sheep; Jackson ImmunoResearch) secondary antibodies diluted in TBS-T at RT for 1 hour. Lastly, the sections were mounted on gelatatin-coated slides after rinsing three times for 10 minutes with TBS-T and secured with coverslips using anti-fading polyvinyl alcohol mounting medium for immunofluorescence imaging (PVA-DABCO, Sigma-Aldrich).

To analyze the transgene expression in TH cells we employed confocal microscopy. The imaging was carried out with Nikon Eclipse Ti-E inverted laser scanning microscope (Nikon, Instruments Inc., Melville, NY) using 488 and 647 nm Sapphire laser lines for excitation of DyLight 488 and Cy5, respectively. The images were collected as Z-stacks using Plan Apochromat 63x N.A. 1.40 oil immersion objective (Nikon). The data was acquired in single channel mode using Nikon EZ-C1 imaging software (v. 3.90), then imported to ImageJ (v. 1.48u4; NIH) and presented as orthogonal projection images.

### BAT staining

For the BAT fat staining, the material was collected from the interscapular area, and the tissue was cut into 7 μm sections at −20 °C with a Microm HM650 microtome (Thermo Scientific). The freshly cut sections from BAT of uninjected, rAAV5-853HTT-18Q and rAAV5-853HTT-79Q mice were stained using Oil Red O Stain Kit for fat (Nordic Biosite) according to manufacturers instructions.

### Metabolic tests

The body weight was followed with bimonthly measurements for 12 months post-injection. The body fat content was determined at 12 months of age using Lunar PIXImus2 dual energy x-ray absorptiometry (DEXA) scanner (Lunar Corporation, USA). A blood sample was collected from the heart left ventricle of animal anesthetized with pentobarbital and the serum was isolated for the assessment of leptin, insulin and IGF-1 levels. The collected blood was kept at room temperature for 30 minutes to clot and the tubes were centrifuged at 2500 × g for 15 minutes for the separation of serum. The collected serum (supernatant) was stored at −80 **°**C in aliquots until further use. Blood serum systemic levels of leptin, insulin and IGF-1 were determined with ELISA (Millipore, Crystal Chem Inc. and Demeditec, respectively) according to the manufacturer’s instructions.

### Gene expression analysis

Gene expression analysis was performed on the hypothalamic and BAT tissue harvested promptly after decapitation. Tissue dissection was carried on ice to minimize the RNA degradation. The collected samples were snap frozen in liquid nitrogen and kept at −80 **°**C for further processing. RNA was isolated using the RNeasy Lipid Tissue Kit (Qiagen) and the reverse transcriptase reaction was performed on 1 μg of sample RNA with SuperScript III Reverse Transcriptase kit (Invitrogen) according to the manufacturers instructions. The gene expression levels were assessed with SYBR Green-based assay (SYBR Green I Master, Roche) with a two-step cycling protocol using LightCycler 480 (Roche). The comparative ΔCt method (ΔΔCT method) was employed to analyze the alterations in gene expression changes relative to glyceraldehyde 3-phosphate dehydrogenase (GAPDH) and β-actin housekeeping genes. The primer sequences are as follows; Y1: Forward-5′-GGCGTTCAAGGACAAGTAT-3′, Reverse-5′-GGAGGAGAGTCGTGTAAGA-3′; TH: Forward -5′-TTCTCAACCTGCTCTTCT-3′, Reverse -5′-TGGCTTCAAATGTCTCAAA-3′; UCP1: Forward -5′-GGTCGTGAAGGTCAGAAT-3′, Reverse -5′-AGAGTTATAGCCACCACAG-3′; GLUT4: Forward -5′-AGTATGTTGCGGATGCTA-3′, Reverse -5′-TTCTTCATCTTCACCTTCCTA-3′; PGC-1α: Forward -5′-AACAATAACAACAACAACCATAC-3′, Reverse -5′-CTGAAGAGGCAAGAGACA-3′; PPARγ: Forward -5′-GCATCAGGCTTCCACTAT-3′, Reverse -5′-GAAGAACCATCCGATTGAAG-3; ADRB3: Forward -5′-CTCCTCACTATGGCTCTC-3′, Reverse -5′-ACAGTTAGGACTTCAAGGT-3; β-actin: Forward -5′-GCTGTGCTATGTTGCTCTA-3′, Reverse -5′-TCGTTGCCAATAGTGATGA-3′; GAPDH: Forward -5′-AACCTGCCAAGTATGATGA-3′, Reverse -5′-GGAGTTGCTGTTGAAGTC-3′; NPY: Forward -5′-CGACACTACATCAATCTCATCA-3′, Reverse -5′-TCTGTGCTTTCCTTCATTAAGAG-3′; HTT: Forward -5′-AGTCAGATGTCAGGATGG-3′, Reverse -5′-CTGTAACCTTGGAAGATTAGAA-3′

### Statistical analysis

To determine the statistically significant differences (p < 0.05) the data was tested for normal distribution using a Kolmogorov–Smirnov test and subsequently with Kruskal–Wallis followed by Dunn’s multiple-comparison test; or one- or two-way ANOVA followed by Tukey’s multiple-comparison test; or unpaired t-test using Prism 6 software (GraphPad). The type of test used for each respective experiment is stated in results section and presented in supplemental statistical results. Data are presented as mean ± SEM.

## Additional Information

**How to cite this article**: Soylu-Kucharz, R. *et al.* Hypothalamic overexpression of mutant huntingtin causes dysregulation of brown adipose tissue. *Sci. Rep.* **5**, 14598; doi: 10.1038/srep14598 (2015).

## Supplementary Material

Supplementary Information
