# Supplementary material for: Hypothalamic overexpression of mutant huntingtin causes dysregulation of brown adipose tissue
Source: Sci Rep. 2015 Sep 30;5:14598. doi: 10.1038/srep14598 (PMC4588570; doi:10.1038/srep14598)
Supplement: Supplementary Information [file srep14598-s1.pdf]

Hypothalamic overexpression of mutant huntingtin causes dysregulation of brown adipose tissue

Rana Soylu-Kucharz[1*], Natalie Adlesic[1], Barbara Baldo[1], Deniz Kirik[2], Åsa Petersén[1]

1. Translational Neuroendocrine Research Unit, Department of Experimental Medical Sciences, Lund University, Sweden
2. Brain Repair and Imaging in Neural Systems (B.R.A.I.N.S.) Unit, Department of Experimental Medical Sciences Lund University, Sweden

Supplementary Statistical Results

Statistical analyses were performed using Prism 6 software (GraphPad) and p<0.05 was considered statistically significant. The data was tested for normal distribution using a Kolmogorov–Smirnov test. M: mean, SD: standard deviation.

Figure 1G: Stereological estimation of TH+ cells in the A13 area.

6 weeks: Unpaired t test with equal SD; t(10)=2.364, p=0.0397(2-tailed)
rAAV5-HTT853-18Q: M=87, SD=12.12, n=6
rAAV5-HTT853-79Q: M=63.5, SD=21.12, n=6

12 weeks: Unpaired t test with equal SD; t(6)=2.455, p=0.0495 (2-tailed)
rAAV5-HTT853-18Q: M=109.3, SD=36.55, n=4
rAAV5-HTT853-79Q: M=57.25, SD=21.41, n=4

18 weeks: Unpaired t test with equal SD; t(6)=4.295, p=0.0051 (2-tailed)
rAAV5-HTT853-18Q: M=100.3, SD=5.795, n=4
rAAV5-HTT853-79Q: M=44.5, SD=25.3, n=4

Figure 2H: Number of TH+ cells in A13 area of the hypothalamus at 12 months post-injection.

One-way ANOVA, F (3, 32) = 16.64, P < 0.0001

Tukey's multiple comparisons test
Uninjected vs. rAAV5-GFP: p=0.2609
Uninjected vs. rAAV5-HTT853-18Q: p=0.3862
Uninjected vs. rAAV5-HTT853-79Q: p= 0.0007
rAAV5-GFP vs. rAAV5-HTT853-18Q: p=0.0031
rAAV5-GFP vs. rAAV5-HTT853-79Q: p< 0.0001
rAAV5-HTT853-18Q vs. rAAV5-HTT853-79Q: p=0.031

Figure 2I: Number of TH+ cells in A14 area of the hypothalamus at 12 months post-injection.

Kruskal-Wallis test: p= 0.0125

Uninjected: M=2756, SD=996, n=7
rAAV5-GFP: M=4658, SD=1634, n=11
rAAV5-HTT853-18Q: M=4300, SD=1344, n=9
rAAV5-HTT853-79Q: M=2852, SD=1369, n=9

Dunn's multiple comparisons test

Uninjected vs. rAAV5-GFP: P=0.0716
Uninjected vs. rAAV5-HTT853-18Q: p=0.2679
Uninjected vs. rAAV5-HTT853-79Q: p> 0.9999
rAAV5-GFP vs. rAAV5-HTT853-18Q: p> 0.9999
rAAV5-GFP vs. rAAV5-HTT853-79Q: p=0.0578
rAAV5-HTT853-18Q vs. rAAV5-HTT853-79Q: p=0.2504

Figure 2J: Number of TH+ cells in A12 area of the hypothalamus at 12 months post-injection.

One-way ANOVA, F (3, 32) = 2.780, P = 0.0569

Tukey's multiple comparisons test

Uninjected vs. rAAV5-GFP: p=0.0758
Uninjected vs. rAAV5-HTT853-18Q: p=0.4135
Uninjected vs. rAAV5-HTT853-79Q: p=0.9727
rAAV5-GFP vs. rAAV5-HTT853-18Q: p=0.7737
rAAV5-GFP vs. rAAV5-HTT853-79Q: p=0.1376
rAAV5-HTT853-18Q vs. rAAV5-HTT853-79Q: p=0.6252

Figure 3J: Number of orexin+ cells in the hypothalamus at 12 months post-injection.

One-way ANOVA, F (3, 30) = 74.63, P < 0.0001

Tukey's multiple comparisons test

Uninjected vs. rAAV5-GFP: p=0.9995
Uninjected vs. rAAV5-HTT853-18Q: p=0.0006
Uninjected vs. rAAV5-HTT853-79Q: p< 0.0001
rAAV5-GFP vs. rAAV5-HTT853-18Q: p=0.0004
rAAV5-GFP vs. rAAV5-HTT853-79Q: p < 0.0001
rAAV5-HTT853-18Q vs. rAAV5-HTT853-79Q: p < 0.0001

Figure 3K: Body weight changes over 12 months.

Two-way ANOVA; Vector: F (3, 345) = 145.5, P < 0.0001
Time: F (4, 345) = 114.2, P < 0.0001
Vector*Time: F (12, 345) = 12.12, P < 0.0001

Tukey's multiple comparisons test

0 months post-injection

Uninjected vs. rAAV5-GFP: p=0.9998
Uninjected vs. rAAV5-HTT853-18Q: p> 0.9999
Uninjected vs. rAAV5-HTT853-79Q: p=0.9658
AAV5-GFP vs. rAAV5-HTT853-18Q: p=0.9998
AAV5-GFP vs. rAAV5-HTT853-79Q: p=0.9472
rAAV5-HTT853-18Q vs. rAAV5-HTT853-79Q: p=0.9665

3 months post-injection

Uninjected vs. rAAV5-GFP: p=0.9956
Uninjected vs. rAAV5-HTT853-18Q: p=0.7224
Uninjected vs. rAAV5-HTT853-79Q: p < 0.0001
rAAV5-GFP vs. rAAV5-HTT853-18Q: p=0.581
rAAV5-GFP vs. rAAV5-HTT853-79Q: p < 0.0001
rAAV5-HTT853-18Q vs. rAAV5-HTT853-79Q: p < 0.0001

6 months post-injection

Uninjected vs. rAAV5-GFP: p=0.9974
Uninjected vs. rAAV5-HTT853-18Q: p=0.3157
Uninjected vs. rAAV5-HTT853-79Q: p < 0.0001
rAAV5-GFP vs. rAAV5-HTT853-18Q: p=0.4189
rAAV5-GFP vs. rAAV5-HTT853-79Q: p < 0.0001
rAAV5-HTT853-18Q vs. rAAV5-HTT853-79Q: p < 0.0001

9 months post-injection

Uninjected vs. rAAV5-GFP: p=0.6717
Uninjected vs. rAAV5-HTT853-18Q: p=0.0504
Uninjected vs. rAAV5-HTT853-79Q: p < 0.0001
rAAV5-GFP vs. rAAV5-HTT853-18Q: p=0.0021
rAAV5-GFP vs. rAAV5-HTT853-79Q: p < 0.0001
rAAV5-HTT853-18Q vs. rAAV5-HTT853-79Q: p < 0.0001

12 months post-injection

Uninjected vs. rAAV5-GFP: p=0.5742
Uninjected vs. rAAV5-HTT853-18Q: p=0.0013
Uninjected vs. rAAV5-HTT853-79Q: p < 0.0001
rAAV5-GFP vs. rAAV5-HTT853-18Q: p < 0.0001
rAAV5-GFP vs. rAAV5-HTT853-79Q: p < 0.0001
rAAV5-HTT853-18Q vs. rAAV5-HTT853-79Q: p=0.3096

Tukey's multiple comparisons test

rAAV5-HTT853-79Q @6 months vs rAAV5-HTT853-79Q @12 months: p=0.005

Figure 3K: DEXA scan measurement at 12 months post-injection.

One-way ANOVA, F (3, 57) = 15.66, P < 0.0001

Tukey's multiple comparisons test

Control vs. rAAV5-GFP: p=0.8945
Control vs. rAAV5-HTT853-18Q: p=0.0046
Control vs. rAAV5-HTT853-79Q: p< 0.0001
rAAV5-GFP vs. rAAV5-HTT853-18Q: p=0.0005
rAAV5-GFP vs. rAAV5-HTT853-79Q: p< 0.0001
rAAV5-HTT853-18Q vs. rAAV5-HTT853-79Q: p=0.7836

Figure 3M: Serum leptin measurement at 12 months post-injection.

One-way ANOVA, F (3, 35) = 20.59, P < 0.0001

Tukey's multiple comparisons test

Uninjected vs. rAAV5-GFP: p=0.8973
Uninjected vs. rAAV5-HTT853-18Q: p=0.0004
Uninjected vs. rAAV5-HTT853-79Q: p < 0.0001
rAAV5-GFP vs. rAAV5-HTT853-18Q: p < 0.0001
rAAV5-GFP vs. rAAV5-HTT853-79Q: p < 0.0001
rAAV5-HTT853-18Q vs. rAAV5-HTT853-79Q: p=0.7414

Figure 3N: Serum insulin measurement at 12 months post-injection.

One-way ANOVA, F (3, 35) = 14.07, P < 0.0001

Tukey's multiple comparisons test

Uninjected vs. rAAV5-GFP: p=0.9998
Uninjected vs. rAAV5-HTT853-18Q: p=0.9205
Uninjected vs. rAAV5-HTT853-79Q: p < 0.0001
rAAV5-GFP vs. rAAV5-HTT853-18Q: p=0.9442
rAAV5-GFP vs. rAAV5-HTT853-79Q: p < 0.0001
rAAV5-HTT853-18Q vs. rAAV5-HTT853-79Q: p=0.0002

Figure 3O: Serum IGF-1 measurement at 12 months post-injection.

One-way ANOVA, F (3, 35) = 4.112, P = 0.0134

Tukey's multiple comparisons test

Uninjected vs. rAAV5-GFP: p=0.6479
Uninjected vs. rAAV5-HTT853-18Q: p=0.2395
Uninjected vs. rAAV5-HTT853-79Q: p=0.3962
rAAV5-GFP vs. rAAV5-HTT853-18Q: p=0.0211
rAAV5-GFP vs. rAAV5-HTT853-79Q: p=0.0428

rAAV5-HTT853-18Q vs. rAAV5-HTT853-79Q: p=0.9828

Figure 4A: Gene expression analysis of the hypothalamus at 8 weeks post-injection.

Y1: One-way ANOVA, F (2, 18) = 10.31, P = 0.0010

Tukey's multiple comparisons test
Uninjected vs. rAAV5-HTT853-18Q: p=0.5429
Uninjected vs. rAAV5-HTT853-79Q: p=0.0011
rAAV5-HTT853-18Q vs. rAAV5-HTT853-79Q: p=0.0109

TH: One-way ANOVA, F (2, 18) = 9.642, P = 0.0014

Tukey's multiple comparisons test
Uninjected vs. rAAV5-HTT853-18Q: p=0.3029
Uninjected vs. rAAV5-HTT853-79Q: p=0.0011
rAAV5-HTT853-18Q vs. rAAV5-HTT853-79Q: p=0.0301

NPY: One-way ANOVA, F (2, 18) = 5.114, P = 0.0174

Tukey's multiple comparisons test
Uninjected vs. rAAV5-HTT853-18Q: p=0.1463
Uninjected vs. rAAV5-HTT853-79Q: p=0.0141
rAAV5-HTT853-18Q vs. rAAV5-HTT853-79Q: p=0.4757

Figure 4B: HTT expression in the hypothalamus at 8 weeks post-injection.

Unpaired t test; t(12)=0.3891, p=0.704 (2-tailed)
rAAV5-HTT853-18Q: M=1, SD=0.2818, n=7
rAAV5-HTT853-18Q: M=1.044, SD=0.106, n=7

Figure 4F: Gene expression analysis of the BAT at 18 weeks post-injection in animals injected with AAV5-HTT853-18Q or AAV-HTT853-79Q vectors in the hypothalamus.

UCP1: Kruskal-Wallis test, p=0.0004
Uninjected: M=1, SD=0.257, n=6
rAAV5-HTT853-18Q: M=1.042, SD=0.05707, n=6
rAAV5-HTT853-79Q: M=0.475, SD=0.1557, n=6
Dunn's multiple comparisons test
Uninjected vs. rAAV5-HTT853-18Q: p> 0.9999

Uninjected vs. rAAV5-HTT853-79Q: p=0.0205
rAAV5-HTT853-18Q vs. rAAV5-HTT853-79Q: p=0.0051

GLUT4: Kruskal-Wallis test, p=0.0088
Uninjected: M=0.9983, SD=0.1158, n=6
rAAV5-HTT853-18Q: M=0.9233, SD=0.3336, n=6
rAAV5-HTT853-79Q: M=0.5767, SD=0.2117, n=6
Dunn's multiple comparisons test
Uninjected vs. rAAV5-HTT853-18Q: p=0.7985
Uninjected vs. rAAV5-HTT853-79Q: p=0.0121
rAAV5-HTT853-18Q vs. rAAV5-HTT853-79Q: p=0.2338

PGC-1α: Kruskal-Wallis test, p=0.0005
Uninjected: M=1, SD=0.1399, n=6
rAAV5-HTT853-18Q: M=1.093, SD=0.2705, n=6
rAAV5-HTT853-79Q: M=0.945, SD=0.2107, n=6
Dunn's multiple comparisons test
Uninjected vs. rAAV5-HTT853-18Q: p> 0.9999
Uninjected vs. rAAV5-HTT853-79Q: p=0.0161
rAAV5-HTT853-18Q vs. rAAV5-HTT853-79Q: p=0.0067

PPARγ: Kruskal-Wallis test, p=2736
Uninjected: M=1, SD=0.1399, n=6
rAAV5-HTT853-18Q: M=1.093, SD=0.2705, n=6
rAAV5-HTT853-79Q: M=0.945, SD=0.2107, n=6
Dunn's multiple comparisons test
Uninjected vs. rAAV5-HTT853-18Q: p> 0.9999
Uninjected vs. rAAV5-HTT853-79Q: p=0.7004
rAAV5-HTT853-18Q vs. rAAV5-HTT853-79Q: p=0.3488

ARDB3: Kruskal-Wallis test, p=0.1425
Uninjected: M=1.002, SD=0.4014, n=6
rAAV5-HTT853-18Q: M=0.8367, SD=0.2384, n=6
rAAV5-HTT853-79Q: M=0.59, SD=0.264, n=6
Dunn's multiple comparisons test
Uninjected vs. rAAV5-HTT853-18Q: p> 0.9999
Uninjected vs. rAAV5-HTT853-79Q: p=0,1439
rAAV5-HTT853-18Q vs. rAAV5-HTT853-79Q: p=0,9097
